# Lactate in Amniotic Fluid: Predictor of Labor Outcome in Oxytocin-Augmented Primiparas’ Deliveries

**DOI:** 10.1371/journal.pone.0161546

**Published:** 2016-10-26

**Authors:** Eva Wiberg-Itzel, Andrea B. Pembe, Hans Järnbert-Pettersson, Margareta Norman, Anna-Carin Wihlbäck, Irene Hoesli, Monya Todesco Bernasconi, Elie Azria, Helena Åkerud, Elisabet Darj

**Affiliations:** 1 Department of Clinical Science and Education, Karolinska Institute, Soder Hospital, Stockholm, Sweden; 2 Muhimbili University of Health and Allied Sciences, Dar es Salaam, Tanzania; 3 Karolinska Institute, Danderyd Hospital, Stockholm, Sweden; 4 Umea University, Umea, Sweden; 5 University of Basel, Basel, Switzerland; 6 Kantonsspital, Aarau, Switzerland; 7 Hospital Bichat Claude Bernard, Paris, France; 8 KBH, Uppsala University, Uppsala, Sweden; 9 Norwegian University of Sciences and Technology, Trondheim, Norway; Shanghai Jiao Tong University, CHINA

## Abstract

**Background:**

One of the major complications related to delivery is labor dystocia, or an arrested labor progress. Many dystocic deliveries end vaginally after administration of oxytocin, but a large numbers of women with labor dystocia will undergo a long and unsafe parturition. As a result of the exertion required in labor, the uterus produces lactate. The uterine production of lactate is mirrored by the level of lactate in amniotic fluid (AFL).

**Objectives:**

To evaluate whether the level of AFL, analysed in a sample of amniotic fluid collected vaginally at arrested labor when oxytocin was needed, could predict labor outcome in nulliparous deliveries.

**Methods:**

A prospective multicentre study including 3000 healthy primiparous women all with a singleton pregnancy, gestational age 37 to 42 weeks and no maternal /fetal chronic and/or pregnancy-related conditions. A spontaneous onset of labor, regular contractions and cervical dilation ≥ 3 cm were required before the women were invited to take part in the study.

**Results:**

AFL, analysed within 30 minutes before augmentation, provides information about delivery outcome. Sensitivity for an acute cesarean section according to high (≥10.1mmol/l) or low (< 10.1mmol/l) AFL values was 39.0% (95% CI; 27–50), specificity 90.3% (95% CI; 87–93) PPV 37.3% (95% CI; 27–48) and NPV was 91.0% (95% CI; 88–93). The overall percentage of correct predictions of delivery outcome when the AFL level was used was 83.7%. Deliveries with a high AFL-level correlated with delivery time >12h (p = 0.04), post-partum fever (>38°C, p = 0.01) and post-partum haemorrhage >1.5L (p = 0.04).

**Conclusion:**

The AFL is a good predictor of delivery outcome in arrested nulliparous deliveries. Low levels of AFL may support the decision to continue a prolonged vaginal labor by augmentation with oxytocin. A high level of AFL correlates with operative interventions and post-partum complications.

## Introduction

Women are suffering high levels of morbidity as a result of a prolonged labor [1–5]. The most common solution for a dystocic delivery is a caesarean section. This method of delivery raises concerns not only regarding risks of complications for the current delivery, but also for future deliveries [6–8]. A more restrictive approach is therefore desirable, especially in deliveries of nulliparous women.

For labor to end successfully, the uterus needs to produce strong, coordinated and effective contractions. Under exertion, lactic acid is produced by glycolysis in all human cells. Glycolysis mainly occurs under hypoxic conditions; but the uterus is highly glycolytic and produces lactate even under normoxic conditions [9–11]. Repeated transient hypoxia is a normal feature of labor as the uterine vessels are occluded during each contraction [9–11]. Lactate will be produced and the levels of pH in the tissue will decrease. Decreasing pH leads to intracellular acidification and an inhibition of the Ca^2+^ channels in the myometrial cells [12]. A decreased inflow of Ca^2+^ into the muscle means that the contraction will be weaker and consequently less effective [9, 10]. New studies show a significant correlation between lactate production, hypoxia and the effect of oxytocin [13–15]. This is an important interaction if labor is to end normally. The level of lactate has been recognized as a factor in uterine activity and pathophysiological processes during labor [13–15].

A partogram should be used to facilitate early detection of poor progress of labor [16–20]. If poor progress is confirmed, oxytocin is suggested for augmentation. Indeed, responses to the administration of oxytocin are variable, and many labors diagnosed as dystocic will be long, painful and unsafe, as the use of oxytocin does not guarantee vaginal delivery, i.e. labor dystocia occurs despite adequate stimulation with oxytocin. A reasonable assumption is that increased knowledge of uterine activity and pathophysiological processes will be the key to the ability to improve the treatment of dysfunctional labors [13–15].

A close correlation has been described between lactate produced by the uterine unit and the level of lactate in amniotic fluid (AFL) [21]. High levels of AFL are shown to be over-represented in dystocic deliveries compared to deliveries with a normal progress of labor [21–25]. A new, non-invasive method has been developed that makes it possible to detect the AFL level in a small sample of amniotic fluid collected from the vaginal pouch and analysed at the bedside in the delivery room. Levels of AFL can be measured immediately and an answer provided within 15 seconds [21–25].

The aim of this study was to evaluate whether the AFL-level, analysed in a sample of amniotic fluid collected at arrested labor when oxytocin was needed, could predict labor outcome in nulliparous deliveries.

## Method

**Fig 1:**
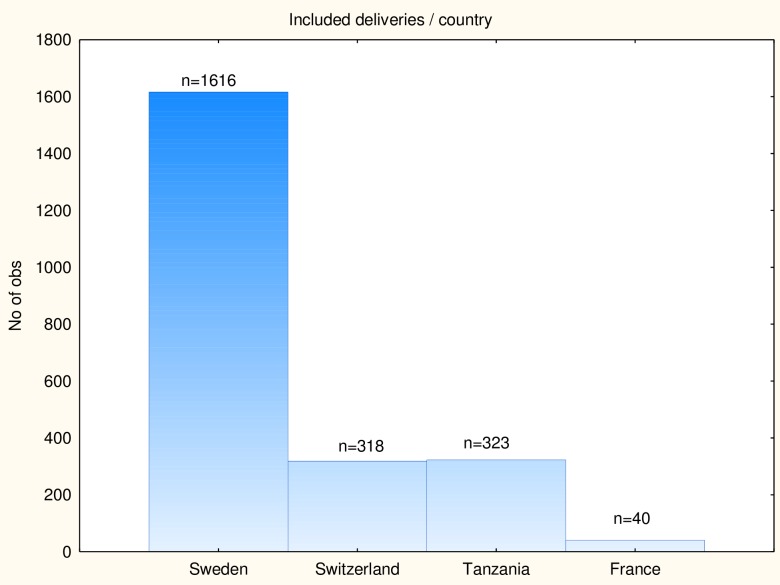


**Fig 2:**
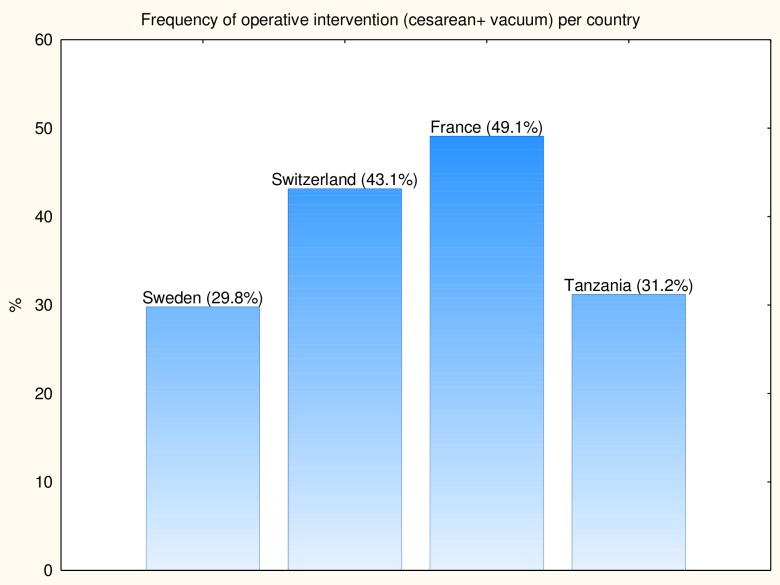


A prospective multicentre trial was performed in delivery wards in Sweden, Switzerland, France and Tanzania between 2010 and 2014. Frequencies of included deliveries and operative interventions per country are presented in Figs 1 and 2.

**Fig 3:**
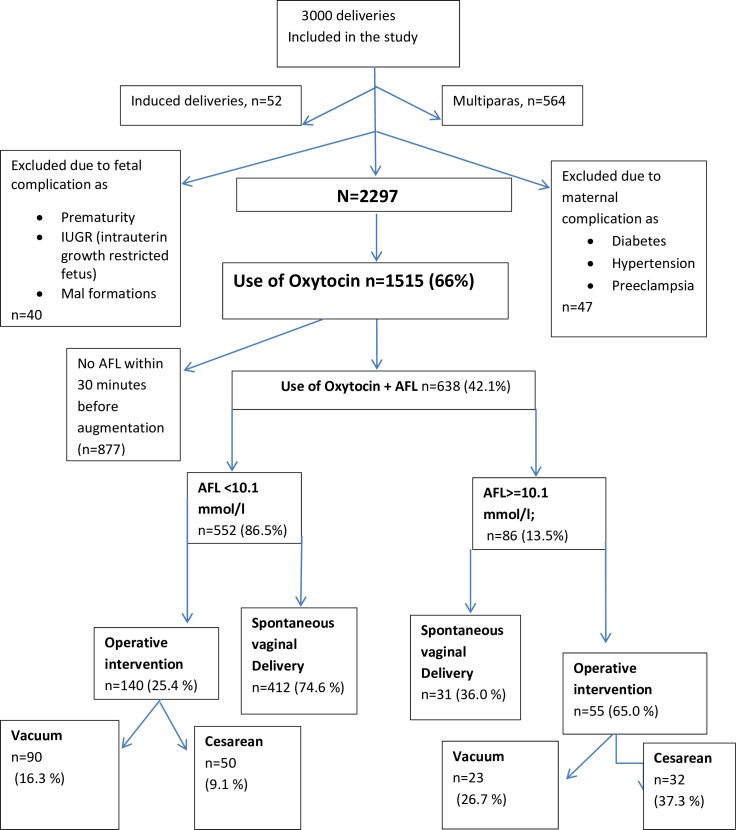


Nulliparous women in active labor at the selected study locations were invited to take part in the study. Inclusion criteria were: a singleton pregnancy, gestational age between 37–42 weeks, no maternal /fetal chronic and/or pregnancy-related conditions. A spontaneous onset of labor, regular contractions and cervical dilation of at least 3 cm were required before the women were invited to take part in the study. A group of midwives and medical doctors at each clinic assisted with data collection and with enrolling women into the study. For all included deliveries a partogram was recorded to monitor the progress of labor. Included clinics used different alert lines of the partogram, but two criteria for labor dystocia were defined: if cervical dilation crossed the action line in the partogram or if labor progress was arrested for two hours or more. Oxytocin was administered according to local clinical guidelines, but the amount of oxytocin added to the infusion did not differ between clinics. A flowchart of the study design is presented in Fig 3.

Obstetric background data, delivery data and AFL values from all deliveries included were reported in a shared internet database completed by a local research midwife. The database was password protected and encrypted. All personal data was encoded, so that individuals could not be identified in the analysis.

Three small samples (50ul) of amniotic fluid (AF) were collected: at the first vaginal examination or when the membranes had been broken, before augmentation with oxytocin and at the time of delivery. If no spontaneously flowing AF was available, a small single-use catheter was used. Previous studies have shown no difference in the value of AFL if AF was flowing spontaneously or collected through a catheter [22]. Amniotomy for augmentation was permitted according to the protocol of the hospital, but not for the sole purpose of obtaining AF for the study.

A device (LMU061, ObsteCare AB, Sweden) adapted for measurement of lactate in amniotic fluid, automatically calibrated at measurement to ensure optimal measurements performance, was available in every delivery ward of the participating hospitals [22]. The device measured lactate concentration in AF with a coefficient of variation of approximately 3% at a lactate concentration of 11mmol/l. The lactate recognition system was based on lactate oxidase with amperometric detection of the enzymatically produced hydrogen peroxide [22].

To investigate the influence of meconium in AF, 10 mL of non-meconium-stained AF was mixed with 2, 4, or 6 mL of meconium. The concentration of lactate in AF was then analysed at regular intervals. The analysed levels of lactate were approximately the same throughout the experiment [22]. Similar tests were performed with a mixture of blood and amniotic fluid. The test showed that the concentration of lactate in AF could be affected by a high concentration of blood. Amniotic fluid samples tinged with a high degree of blood (≥ 10%) have therefore been removed from the analysis.

The AFL was analysed immediately by a research midwife. The result was delivered within 15 seconds, blinded and stored in the device, and not communicated until after delivery. The Apgar score at 1 and 5 minutes after delivery was determined by the midwife in charge.

A monthly quality check of the lactate measurement devices used in the study was made by an external company (Equalis AB, Uppsala, Sweden) that sent an unknown standard solution of lactate for analysis to all included delivery wards. The results were compiled for all analysers and presented monthly by the company. No significant differences were shown between the analytic results of the devices during study time.

### Statistical analysis

To detect a difference of at least 15 percentage units in the proportions of cesaeren sections between high/low AFL levels, with 83% power, we needed to include 160 deliveries with low (< 10.1 mmol/l) and 80 with high (≥ 10.1 mmol/l) AFL values. We assumed that at least 50% of the women included would receive oxytocin, and of these 15% would have a high AFL value, which implied that a minimum of 1444 (160/0.85+80/0.15)/0.5) deliveries had to be included in the study. The recruitment goal was then set at 3000 to allow for missing data or missing AFL samples. Differences among groups defined by operative delivery were tested by ANOVA for continuous variables and chi-square tested for categorical variables. The cut-off value of AFL was set at ≥10.1 mmol/L. This was in line with earlier publications where an increased frequency of operative intervention has been seen in deliveries with an AFL value ≥ 10.1 mmol/L [22–24].To evaluate the predictive capabilities of AFL, sensitivity, specificity, positive (PPV), negative (NPV) predictive values for operative intervention were calculated with 95% confidence intervals (CI). Logistic regression was used to study the association between operative interventions and each of the independent factors: maternal age, education, gestational age, fetal presentation, latent phase duration, use of epidural anesthesia (EDA), AFL, and country of delivery [25]. Our model strategy was as follows: first, unadjusted associations with each factor were studied; second, the adjusted association with respect to the risk factors measured was studied in a multivariable model with all factors included; third, a subgroup analysis was performed to study whether the operative interventions differed between those with high and low AFL with respect to the levels of the other factors. An interaction term between AFL and each of the factors was added to the adjusted model (sequentially). P-values < 0.05 were regarded as statistically significant. Analyses were performed using IBM SPSS 22 (SPSS Inc. Chicago, Illinois, USA).

The study was approved by the regional ethics committee at Karolinska Institutet, Stockholm (2010/199-31/1). All the clinics involved added a local addendum from their ethics committee to the original ethical authorisation. Written informed consent was obtained from all the women before inclusion in the study.

## Results

A total of 3000 deliveries were included in the study. However, 703 mostly African women were subsequently excluded due to failure to meet the inclusion criteria. This was something detected after enrolment, and most common reason was that they were not primiparas or was not completely healthy (Fig 3, flow chart). Table 1 describes demographic data for the entire study population according to nationality. The African women were younger and less educated than the other participants; their pregnancies were shorter, and African new-borns were smaller at delivery.

**Table 1:** Maternal and fetal background data presented per country for deliveries with a sample of amniotic fluid collected at the time of labor. Data are presented as numbers (%) or mean (SD) n = 2049.

Characteristic	Sweden (n = 1415)	Switzerland (n = 278)	France (n = 40)	Tanzania (n = 316)	P-value*
**Maternal**					
Age (years)	30.6 (4.8)	29.8 (4.7)	29.3 (4.1)	24.0 (5.2)	<0.001*
Smoker (%)	200 (14.3)	42 (15.9)	9 (22.5)	Missing ^	0.4
Education High** (%)	1302 (92.0)	204 (73.4)	35 (89.7)	42 (13.3)	<0.001*
**Fetal**					
Gestational age (days)	281 (7.3)	279 (7.0)	279 (6.3)	273 (8.8)	<0.001*
Gender of the fetus (Boys %)	712 (50.4)	138 (49.6)	23 (59.0)	164 (51.9)	0.9
Fetal weight (g)	3592 (445)	3394 (410)	3410 (377)	3085 (406)	<0.001*
Fetal height (cm)	51.0 (1.9)	50.1 (2.0)	50.3 (1.7)	Missing ^	<0.001*
Head circumference (cm)	35.0 (1.4)	34.9 (1.3)	34.8 (1.3)	Missing ^	0.6
Presentation (Occiput posterior %)	89 (6.3)	49 (17.7)	1 (2.6)	20 (9.2)	<0.001*

*P -Values<0.05 were considered statistically significant

^ Not presented in Tanzanian deliveries

** High education was defined college or more

Oxytocin was used in 66% (1515/2297) of all deliveries in the study. Table 2 presents delivery characteristics in the augmented deliveries where an AF sample was collected within 30 minute before oxytocin was administered (n = 638). In 877 deliveries, a sample of AF was not collected within 30 minutes, and they were excluded from the calculation.

**Table 2:** Delivery data presented per high/low AFL value at the time of augumentation. Data are presented as numbers (%), n = 638.

Characteristics	AFL<10.1 mmol/l at augumentation (n = 552)	AFL≥10.1 mmol/l at augumentation (n = 86)	p-value*
Inclusion according to country			0.06
• Sweden • Switzerland • France • Tanzania	• 385 (69.7) • 55 (10.0) • 13 (2.4) • 99 (17.9)	• 69 (80.2) • 4 (4.7) • 4 (4.7) • 9 (10.4)	
AFL when attending delivery ward			<0.001*
• ≥10.1 mmol/l • <10.1 mmol/l • Missing	• 17 (3.1) • 532 (96.9) • 3/552 (0.2)	• 63 (73.3) • 23 (26.7) • 0	
AFL at delivery			<0.001*
• ≥10.1 mmol/l • <10.1 mmol/l • Missing	• 53 (9.7) • 493 (90.3) • 6/552 (1.1)	• 63 (73.3) • 23 (26.7) • 0	
Latent phase duration > 15h			0.5
• Yes • No • Missing	• 92 (16.7) • 459 (83.3) • 1 (0.2)	• 12 (13.9) • 74 (86.1) • 0	
Way of delivery			<0.001*
• Spontaneous vaginal • Vacuum/forceps • Emergency cesarean section	• 412 (74.6) • 90 (16.3) • 50 (9.1)	• 31 (36.0) • 23 (26.7) • 32 (37.3)	
Vacuum/forceps delivery due to			
• Labor dystocia • Fetal distress • Other	• 48 (53.3) • 32 (35.6) • 10 (11.1)	• 18 (78.2) • 5 (21.8) • 0	
Emergency cesarean section due to			
• Labor dystocia • Fetal distress • Other	• 38 (76.0) • 11 (22.0) • 1 (2.0)	• 27 (84.3) • 5 (15.7) • 0	
Labor dystocia according to the partogram			0.008*
• Yes ○ Primary ○ Secondary • No	• 406 (73.6) • 256 (63.5) • 150 (36.5) • 146 (26.4)	• 74 (86.0) • 52 (70.2) • 22 (29.8) • 12 (14.0)	
Active time of delivery > 12h			0.04*
• Yes • No	• 124 (22.5) • 428 (77.5)	• 28 (32.6) • 58 (67.4)	
Use of epidural anaesthesia(EDA)**			0.007*
• Yes • No	• 339 (61.4) • 213 (38.6)	• 65 (75.7) • 20 (23.3)	
Fever >38°C post-delivery			0.01*
• Yes • No • Missing	• 37 (8.1) • 418 (91.9) • 97/552 (22.1)	• 13 (16.9) • 64 (83.1) • 9/86(10.5)	
Post-partum hemorrhage >1.5 L			0.04*
• Yes • No • Missing	• 10 (2.3) • 420 (97.7) • 122/552 (22.1)	• 5 (6.7) • 70 (93.3) • 11/86 (12.8)	
Apgar score < 7 at 5 minutes after delivery			0.5
• Yes • No • Missing	• 13 (2.4) • 536 (97.6) • 3 (0.5)	• 1 (1.2) • 85 (98.8) • 0	
Ph in arterial cord blood < 7.05 at delivery **			0.7
• Yes • No • Missing	• 4 (1.0) • 406 (99.0) • 142/552 (25.7)	• 1 (1.5) • 68 (98.5) • 17/86 (19.8)	

*P -Values<0.05 were considered statistically significant

** Not used in Tanzanian deliveries

In the augmented deliveries the spontaneous vaginal delivery rate was 69.4% (443/638). 75.2% (480/638) of them were diagnosed with labor dystocia according to the partogram, and 64.2% (308/480) of deliveries with labor dystocia arrested at the first stage of labor. 23.8% (152/638) of the augmented deliveries had an active period of labor for more than 12 hours.

Of the new-borns, 2.2% (14/638) had an Apgar score of < 7, at 5 minutes after delivery, and most affected new-borns were seen in Tanzania (3.7% with a low apgar score). One maternal and three fetal deaths occurred in the study, all of them in the Tanzanian group.

Totally, 5418 samples of AF were collected in the study. 1854 of them at the first vaginal examination after the woman attended delivery ward, 1515 prior to augmentation with oxytocin (638 of them within 30 minutes), and 2049 at the time of delivery. Deliveries with AFL ≥ 10.1 mmol/l correlated with an increased frequency of operative intervention (p < 0.001) and with an active time of delivery >12h (p = 0.04). Significantly more epidurals were used in the high AFL group (p = 0.007), and the group also showed a higher incidence of post-partum fever (>38°C, p = 0.01) and post-partum hemorrhage >1.5L (p = 0.04, Table 3).

**Table 3:** Sensitivity, Specificity, Positive (PPV) and Negative Predictive value (NPV) for operative delivery among 638 deliveries where oxytocin was used and an AF sample was collected within 30 minutes before commencing stimulation with oxytocin. The AFL values were used for predictive purposes.

Predicted	Observed		Total
	Cesarean section	Vaginal delivery	
AFL ≥ 10.1 mmol/l	32	54	86
AFL<10.1 mmol/l	50	502	552
Total	82	556	638

Sensitivity = 32/82 = 39.0% (95% CI; 27 to 50)

Specificity = 502/556 = 90.3% (95% CI; 87 to 93)

NPV = 32/86 = 37.3% (95% CI; 27 to 48)

PPV = 502/552 = 91.0% (95% CI; 88 to 93)

Overall percentage of correct predictions: 83.7% (502 + 32 = 534/638 = 83.7%)

According to study design AFL-values within the 30 minutes before augmentation were used in our prediction of labor outcome. The sensitivity for a cesarean section according to high/low AFL values was 39%, the specificity 90%, the PPV 37%, and the NPV was 91%. The overall percentage of correct predictions when AFL was used was 84% (Table 3).

High maternal age, gestational age > 41w, occiput posterior presentation of the fetus, the use of EDA, being delivered in Switzerland and an AFL level ≥10.1 mmol/l at the time of augmentation were all associated with an increased likelihood of operative intervention (Table 4).

**Table 4:** Associations between possible risk factors and the risk of an operative intervention. Values are expressed as Odds Ratio (OR) with corresponding 95% confidence intervals (CI). (n = 638).

*Risk factors for operative intervention during delivery*	OR unadjusted(95% CI)	OR adjusted (95% CI)
**Maternal age:** < 30 years vs. >30 years**	1.9 (1.3 to 2.7)*	1.7 (1.1 to 2.7)*
**Maternal education:** Low ^ vs. High^^**	2.4 (1.5 to 3.7)	0.8 (0.4 to 1.5)
**Gestational age:** <41+0 weeks vs. >41+0 weeks**	1.9 (1.3 to 2.9)*	1.8 (1.1 to 3.0)
**Fetal presentation:** Anterior vs. Posterior	11.6 (5.5 to 24.6)*	9.6 (4.2 to 21.9)*
**Latent phase:** <15h vs. >15h	1.8 (1.2 to 2.7)	1.5 (0.9 to 2.5)
**Arrested labor progress according to the partogram:** No vs. Yes	1.9 (1.3 to 3.0)*	1.7 (1.0 to 3.1)
**Epidural anesthesia:** No vs Yes	3.4 (2.3 to 5.1)*	1.8 (1.1 to 3.1)*
**AFL > 10.1 mmol/l when oxytocin was initiated:** No vs. Yes	5.2 (3.2 to 8.4)*	4.5 (2.6 to 8.1)*
**Countries**: Sweden vs. Switzerland vs. Tanzania vs France	2.3 (1.4 to 4.0)*vs. 0.2 (0.1 to 0.5)*vs. 1.5 (0.6 to 4.0)	4.2 (2.2 to 8.2)* vs. 0.3 (0.1 to 5.1) vs. 1.7 (0.6 to 5.1)

^ Primary school

^^ High school or more

*P values<0.05 were considered as statistically significant

**All data not available from the African part of the study

No significant association was indicated between the levels of education, a long latent phase, or labor dystocia according to the partogram.

High AFL value (≥10.1 mmol/l) and occiput posterior presentation of the fetus had the strongest association with operative intervention after adjusting for the other factors (Table 4). No significant interactions between AFL and each of the other eight factors were detected (p > 0.48 for all test of interactions), which implies that the sensitivity for operative intervention among women with a high AFL value (≥ 10.1 mmol/l) was 4.5 times higher compared to women with a low AFL value, irrespective of the levels of the other factors.

## Discussion

This large observational study of healthy nulliparous women from different countries and settings presents a new way of describing labor dystocia. The lactate values in amniotic fluid analysed just before augmentation with oxytocin provides important information about the uterus. Low levels of AFL may support the decision to continue a prolonged vaginal labor by augmentation with oxytocin as the uterus appears to be receptive to augumentation, whereas high levels of AFL indicate a higher risk not only of cesarean section, but also of post-partum complications.

Labor dystocia is a leading indication for cesarean section worldwide [26–31]. Several publications have linked a number of well-known risk factors for an operative intervention among otherwise low-risk women [29–31]. In this work we have studied some of these risk factors, as well as their relation to the metabolic status of the uterus presented as the AFL value. Our results show that AFL is a new predictor of labor outcome in arrested labors. If a high AFL level is present when augmentation starts, the likelihood of an operative intervention increases 4.5 times irrespective of the levels of the other known risk factors (Table 4).

Another very important observation of this study is that the partogram, recommended by the WHO, did not predict labor outcome. More than half of all deliveries in this study were diagnosed as having labor dystocia according to the partogram, whilst over 60% of them ended in a spontaneously vaginal delivery. Our reflection is that deliveries among primiparas may be different from how they were described by Friedman and his co-workers who created the partogram in the 1950s [18, 19]. Our message is that whilst the partogram is in every way an important tool for keeping records of activity during childbirth, it is not a useful predictor of delivery outcome [16–20].

A study of AFL was just recently performed at Maternity Hospital, Dublin, Ireland, and presented by Murphy et al [32]. In the Dublin study, the first sample of AFL, when a woman attended the delivery ward, was analysed. They concluded that primarily, high levels of lactate in amniotic fluid in spontaneously laboring; single cephalic, nulliparous womans deliveries is a new and independent predictor of labor disorder and cesarean section [32].

Strength of this project is the understanding why some dystocic deliveries, despite adequate stimulation with oxytocin, do not reach a spontaneous vaginal delivery. It is also a strength that clinics from various settings are included, which makes it possible to confirm that the method of AFL is easy to use, irrespective of geography, culture or clinic size. Some limitations of the study should be noted. As it was not feasible to change clinical guidelines simply for this observational study. Different methods of managing arrested labors were seen in the clinics included, even though the same inclusion criteria were used for the study. The number of deliveries eligible to be included was also limited, due to the strict inclusion criteria: all women included had to be healthy nullipara with a spontaneous onset of labor, in order to avoid bias and interference factors.

The main question in this work is whether the introduction of this new method could help obstetricians/midwives to make a more informed decision in the delivery room. When no information about AFL is available, a good guess would be that arrested delivery will end in a vaginal delivery after a proper administration of oxytocin. If that assumption was correct, the overall percentage of correct classifications of labor outcomes in this study would be 69.4% (Table 4). The sensitivity for an operative delivery would be 0% and the specificity 100%. If the measure of AFL had been used together with the partogram, the overall percentage of correct classifications is better (84%). 39% of the women who had an operative intervention at a later stage would have been identified earlier in the process through their raised AFL levels, hopefully with a higher possibility of avoiding a long and painful parturition. 39% can be perceived as a rather low figure, but in clinical obstetrics of today’s, this prediction cannot be made at all.

The predictive values of AFL may be of more interest for the individual obstetrician/midwife. The NPV imply that more than 90% of cases with a low AFL will have a vaginal delivery. This represents important information. We anticipate that the number of unnecessary cesarean sections in this group can be reduce, as important information about the uterine receptivity to augmentation hopefully lead the to a more individually adapted use of oxytocin and to more normal deliveries. The NPV imply that 37% of deliveries with high AFL would end in an operative intervention. This is also important information for the staff in charge. This is a small group, approximately 15% of the material, but shown to have a higher frequency of complications, probably due to the prolonged time of delivery. This is probably the group described earlier where labor dystocia occurs despite adequate stimulation with oxytocin. By using this new method, very long deliveries can be avoided, caesarean sections that will be carried out can be made at an earlier stage, and postpartum complications will hopefully be reduced. The study has confirmed a new means of classifying dystocic deliveries into an early identification of a normal group with a good receptivity of oxytocin and a possibility of a vaginal delivery after an adequate use of oxytocin, and a group where problems are likely to arise. This is valuable information, especially in countries where referral to a higher level of care may be needed.

One important factor to be taken into consideration is how common an operative intervention is in a specific population. This is problematic as little or no common consensus exists, what labor dystocia really is and how to handle a dystocic delivery. Operative interventions in this study are more common in Switzerland; the predictive value of AFL might therefore be better in this subgroup than among the Tanzanian women, where fewer deliveries end in an operative intervention. On the other hand, more complications such as post-partum infections and post-partum hemorrhages are seen among Tanzanian women in the study. One maternal and three fetal deaths occurred in the Tanzanian group, all of them after extremely long deliveries with high AFL values. AFL is likely to be an even more useful measurement in these settings. The question becomes, whether we are using the correct operative delivery criteria when handling a dystocic delivery. Hopefully this study can help us to reach a new kind of consensus with a reduction in the number of unnecessary operative interventions.

**“In conclusion”** we have found that the AFL values collected within 30 minutes of augmentation with oxytocin are an early predictor of labor outcome in dysfunctional primiparous deliveries. Given that today many deliveries are unnecessarily long with risks of increased perinatal morbidity; our findings have important implications for public health.

## Supporting Information

S1 File Dataset A. (XLSX)
